# Single nucleotide polymorphism of *FGF2 *gene in Iranian Holstein proven bulls

**Published:** 2015-01

**Authors:** Abdolreza Salehi, Rohalah Sobhani, Mahdi Aminafshar, Mohammad Bagher Sayyadnejhad, Khadijeh Nasiri

**Affiliations:** 1 Department of Animal and Poultry Science, College of Aburaihan, University of Tehran, Tehran, Pakdasht, Iran.; 2 Department of Animal Science, Faculty of Agriculture and Natural Resources, Science and Research Branch, Islamic Azad University, Tehran, Iran; 3 Animal Breeding Center of Iran, Karaj, Iran

**Keywords:** *FGF2*, SNP, Iranian Holstein, Polymorphism, Candidate gene

## Abstract

Fibroblast growth factor *2 *(*FGF2*) serves in the uterine endometrium during estrous presenting in the bovine mammary gland which is responsible to express interferon-*T *(IFNT), and is an important agent to encourage the continuation of pregnancy in the ruminants. Significant associations have been found between genes affected by IFNT and genes that are responsible for milk production traits. Semen samples from 101Iranian Holstein proven bulls were collected to extract the genomic DNA. Forward and reverse primers were designed and a 710-base-pair fragment in intron 1 was amplified using PCR technique. To detect single nucleotide polymorphism (SNP), all samples were sequenced. Three positions including 11474 (C/G), 11513 (C/G) and 11646 (A/G) were considered. The 11474C, 11513C and 11646A alleles are known as wild type alleles. In this study all animals were distinguished as the11474C, 11513C and 11646A alleles. Furthermore, amplified fragments were under consideration to detect new SNPs. Only one new SNP in one sample was observed at position 11863 resulting substitution of thymine to cytosine. This new mutation has been registered on the NCBI database with accession number HM597774.

## INTRODUCTION

The results of meta-analysis suggest that many QTL have been mapped for economic important traits in dairy cattle [1]. However, despite a number of QTL studies in cattle and other species, little advance has been done on the identification of major genes affecting milk production and health traits in dairy cattle [2]. Researches show that STAT1, UTMP, OPN, FGF2, ABCG2 and triglyceride synthesis Diacylglycerol Acyltransferase 1 (DGAT1) have a major effect on milk yield and compositions in dairy cattle [3-6]. Wang et al., [2] detected one SNP (A/G) in intron 1 at position 11646 that was associated with fat yield and percentage, somatic cell score, and productive life. Seventeen SNPs have been detected within the bovine *FGF2 *gene in introns 1 and 2 and have been offered on the NCBI database.

*FGF2 *gene is located in chromosome 17 between *BBS12 *and *NUDT6 *genes and it is about 59kb in length comprising 3 exons coding a 156-amino-acid protein [2, 7]. Fibroblast growth factor (FGF) family show a wide set of autocrine and paracrine factors, which control several important functions in body [8]. Fibroblast growth factor 2 (FGF2 or basic) is mostly expressed in bovine theca cells [9] and synthesize in the uterine endometrium during the estrous cycle and early pregnancy. The bovine mammary gland also produces FGF [10]. Due to expression of *FGF2 *gene in mammary gland, it may be important for the development and reorganization of the mammary gland [10]. Presence of FGF2 mRNA and FGFR2 (one of FGF2 receptor partners) has been reported throughout early bovine embryo development [11-13].

Several researches have shown that some genes dependent on interferon-*T*(IFNT) and placental lactogen signal transduction pathway are associated with milk production, health, and fertility traits in dairy cattle [5, 14, 15]. FGF2 controls expression of interferon-*T *(IFNT) which is a particular factor to promote continuation of pregnancy in ruminants [2, 6, 7]. IFNT activates Signal Transducer and Activator of Transcription 1(*STAT1*) and *STAT2 *genes, which cause production of uterine milk protein (UTMP) and osteopontin (OPN) [2, 16].

Hence, *FGF2 *gene sounds a good candidate gene affecting milk production traits. Study of single nucleotide polymorphism in the *FGF2 *gene as a candidate gene in Iranian Holstein proven bulls were principal aims of this investigation due to relationship between *FGF2 *gene and IFNT and also effects of IFNT on pregnancy and milk production traits.

## MATERIALS AND METHODS

**DNA preparation: **A total of 101 semen samples from sires were obtained from the progeny tested Iranian Holstein bulls representing resource populations (as a whole Iranian registered Holstein populations). At least 30 daughters per each bull was available. Genomic DNA was extracted from 200µl of semen by using High Pure PCR Template Preparation Kit (Roche Company kit, CAD No=11796828001) along 7µl Dithiothreitol (DTT) per reaction and modified salting-out method. The DNA concentrations were measured via spectrophotometer (PicoDrop, England).

**DNA amplification and SNP Detection: **The primers including Forward: 5ˈ-CTT CAT CCC CTC AGT CTT C-3ˈ and Reverse: 5ˈ-CAC TCA TCT GCT GGT AAC TTC-3ˈ were designed with Oligo (version 7, 2011) to amplify a 710-bp fragment from position 11254 to 11963 in intron 1 of bovine *FGF2 *gene (GenBank accession number NW_001495237). Polymerase chain reaction was performed with 150ng of DNA in a 40µL reaction volume comprising 1x PCR buffer, 200µM dNTP, 1 unit of Taq DNA polymerase, 2 mM MgCl2, 0.4µM of each primer. Temperature cycles were adjusted as follows: Initialization step: 95°C for 150s; pursued by 35 cycles of denaturation: 95°C for 1min; annealing: 58°C for 70s; extension: 72°C for 1min and followed by a final extension step 72°C for 15min. The digestion products were subjected to 1.5% polyacrylamide gel electrophoresis (PAGE) and 1x TBE buffer. In addition, 100-size Ladder was run as molecular weight marker. Eventually, to detect single nucleotide polymorphisms (SNP) in the FGF2 locus, the PCR products were sequenced and then sequenced products were analyzed and aligned by CLC Sequence Viewer software (company: CLC bio A/S, version 6.3.0.0.)

## RESULTS AND DISCUSSION

**Figure 1:**
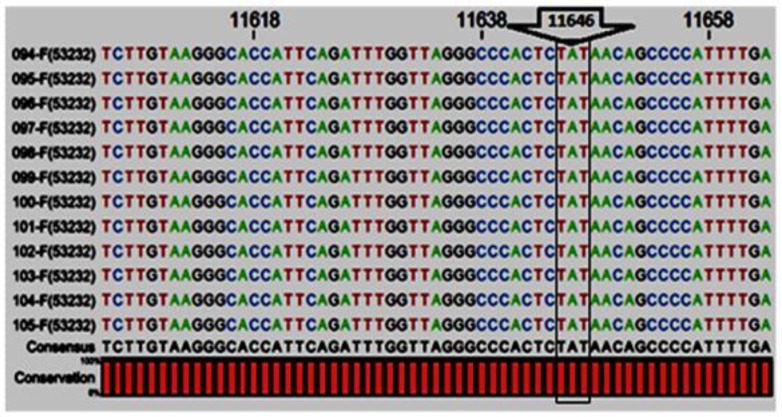
Position 11254 to 11963 in intron 1 of *FGF2* gene. There was no any mutation at 11646 (A/G)

To detect new mutations, a 710-bp fragment between position 11254 and 11963 in intron 1 was amplified and sequenced in order to study effects of 3 SNPs at positions 11474 (C/G), 11513 (C/G) and 11646 (A/G) on milk production traits. The 11474C, 11513C and 11646A are known as wild type alleles, respectively. In this study, all animals were distinguished as the 11474C, 11513C and 11646A alleles and there was no any polymorphism at these positions. Based on this study, all of these positions were shown to be monomorphic (Fig. 1).

position (some samples have been only shown).

**Figure 2:**
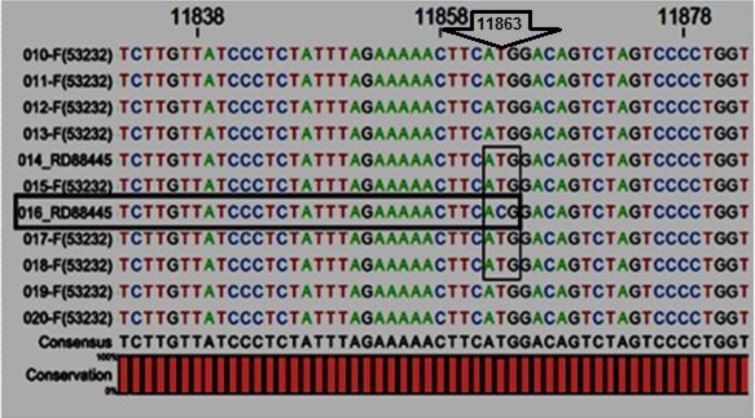
New SNP for *FGF2* locus is located at position 11863 of intron 1 substituting thymine to cytosine.

All of the sequenced products were aligned and analyzed to find out new SNPs. Using the DNA sequencing approach, only 1 T/C SNP was detected, at position 11863in intron 1 of *FGF2 *in one sample changing thymine to cytosine (Fig. 2). Consequently, this new mutation has been registered with accession number HM597774 on the NCBI database.

This study was conducted to investigate the IFNT and placental lactogen signal transduction pathway as a candidate pathway affecting production traits in Iranian dairy cattle population. We assume that candidate genes affecting quantitative traits can be identified by tracking their biological action through intracellular effector pathways. Many studies have shown that several genes of the interferon-τ (IFNT) and placental lactogen signal transduction pathway including STAT1 [13], UTMP [4], OPN [5], STAT5 [17], were associated with milk production, health, and fertility traits in dairy cattle. As FGF2 is a member of the IFNT signal transduction pathway and controls the expression of IFNT [2, 6, 7], which regulates expression of STAT1, STAT2, OPN and UTMP, it was chosen for investigation of association with milk production. In addition, *FGF2 *gene is produced in the mammary gland for developing and reorganizing this tissue. However, there was no any published article on *FGF2 *gene in Iranian Holstein proven bulls.

In investigated population no polymorphism was identified at mentioned positions. Allele A was only identified at position 11646 and was shown to be monomorph in Iranian Holstein proven bulls which is in contrast with the results presented by Wang et al., [2]. They studied association between SNP at position 11646 (A/G) with milk production traits on three populations. They claimed that SNP at position 11646 in one of populations had significant effects on fat yield and fat percentage and frequencies of alleles A and G were 0.35 and 0.65, respectively [2]. In fact, they found allele G is cause of increasing fat yield and fat percentage. In the last two decades, selection was in favor of increasing milk yield in Iranian Holstein population. As there is a negative genetic correlation between milk yield and fat percentage, it seems likely that the pressure has been done on the elimination of the allele G. As only one new mutation (SNP at position 11863) was detected in all samples it was not possible to do the association test for milk production traits, so that no information concerning allelic and genotypic frequencies were reported as well as no data about a possible association with milk production traits. Further studies on effects of other FGF2 locus SNPs (in intron or exon) would yield information facilitating for the milk and reproductive traits for bulls. Moreover, it is essential to confirm new mutation at position 11863 (T/C) on larger population of animals.

In conclusion, a total of 101 individuals as a whole population of proven bull was examined. We detect only one new mutation at position 11863 (T/C) in just one sample, so that the analysis of association of this SNP with production traits was not possible. Therefore, it might be useful to investigate more number of bulls in future. On the other hand, further studies on effects of other FGF2 locus SNPs (in intron or exon) would yield information facilitating for the milk and reproductive traits. Because of attitude to increasing milk yield and close relation of Iranian Holstein with American breed, it is possible that imported semen had only carried allele A. Moreover, it is essential to confirm new mutation at position 11863 (T/C) on larger population of animals.

## Notes

**Acknowledgements: **This research was conducted in the genetic laboratory at the department of animal and poultry science, college of Aburaihan, University of Tehran. Authors would like to thank Ms. Somayyeh Zeynali, Ms. Narjes Mohammadi and Mr. Saeid Momeni for their great cooperation. We also thank the animal breeding center staff for providing semen samples of Iranian Holstein bulls with the great help of Mr. Keikhosro Amini.
